# Diagnostic performance of tomoelastography of the liver and spleen for staging hepatic fibrosis

**DOI:** 10.1007/s00330-019-06471-7

**Published:** 2019-11-11

**Authors:** Rolf Reiter, Heiko Tzschätzsch, Florian Schwahofer, Matthias Haas, Christian Bayerl, Marion Muche, Dieter Klatt, Shreyan Majumdar, Meltem Uyanik, Bernd Hamm, Jürgen Braun, Ingolf Sack, Patrick Asbach

**Affiliations:** 1 Department of Radiology, Charité – Universitätsmedizin Berlin, corporate member of Freie Universität Berlin, Humboldt-Universität zu Berlin, and Berlin Institute of Health, Berlin, Germany; 2 Richard and Loan Hill Department of Bioengineering, University of Illinois at Chicago, Chicago, United States; 3 Medical Department, Division of Gastroenterology, Charité – Universitätsmedizin Berlin, corporate member of Freie Universität Berlin, Humboldt-Universität zu Berlin, and Berlin Institute of Health, Berlin, Germany; 4 Department of Medical Informatics, Charité – Universitätsmedizin Berlin, corporate member of Freie Universität Berlin, Humboldt-Universität zu Berlin, and Berlin Institute of Health, Berlin, Germany

**Keywords:** Elasticity imaging techniques, Magnetic resonance imaging, Liver cirrhosis, Fibrosis, Spleen

## Abstract

**Objectives:**

To determine the diagnostic performance, cut-off values, and optimal drive frequency range for staging hepatic fibrosis using tomoelastography by multifrequency MR elastography of the liver and spleen.

**Methods:**

This prospective study consecutively enrolled a total of 61 subjects between June 2014 and April 2017: 45 patients with chronic liver disease and proven stage of fibrosis and 16 healthy volunteers. Tomoelastography was performed at 1.5 T using six drive frequencies from 35 to 60 Hz. Cut-off values and AUC were calculated. Shear wave speed (in m/s) of the liver and spleen was assessed separately and in combination as a surrogate of stiffness.

**Results:**

For compound multifrequency processing of the liver, cut-off and AUC values by fibrosis stage were as follows: F1, 1.52 m/s and 0.89; F2, 1.55 m/s and 0.94; F3, 1.67 m/s and 0.98; and F4, 1.72 m/s and 0.98. Diagnostic performance of the best single drive frequencies (45 Hz, 55 Hz, 60 Hz) was similar (mean AUC = 0.95, respectively). Combined analysis of the liver and spleen slightly improved performance at 60 Hz in F4 patients (mean AUC = 0.97 vs. 0.95, *p* = 0.03). Full-field-of-view elastograms displayed not only the liver and spleen but also small anatomical structures including the pancreas and major vessels.

**Conclusion:**

Tomoelastography provides full-field-of-view elastograms with unprecedented detail resolution and excellent diagnostic accuracy for staging hepatic fibrosis. Our analysis of single-frequency tomoelastography suggests that scan time can be further reduced in future studies, making tomoelastography easier to implement in clinical routine.

**Key Points:**

*• Tomoelastography provides full-field-of-view elastograms of the abdomen with unprecedented detail resolution and excellent diagnostic accuracy for staging hepatic fibrosis.*

*• Diagnostic performance of single-frequency tomoelastography at higher frequencies (45 Hz, 55 Hz, 60 Hz) and compound multifrequency processing are equivalent for staging hepatic fibrosis.*

*• Combined assessment of hepatic and splenic stiffness slightly improves diagnostic performance for staging hepatic fibrosis.*

## Introduction

Detection and treatment of hepatic fibrosis at an early stage can prevent ongoing hepatocellular damage, progression towards cirrhosis, and complications such as portal hypertension and ascites. The combined examination of the liver and spleen has emerged as a promising field of research [1–4]. It has been shown that, in hepatic fibrosis, not only hepatic stiffness but also splenic stiffness increases, which is presumably caused by an increased pressure in splenic vasculature [1, 4, 5]. However, data regarding the staging of hepatic fibrosis based on mechanical tissue properties of both liver and spleen are still limited, and no investigation aimed at defining the optimal frequency range of elastography has been performed [5].

Magnetic resonance elastography (MRE) is a noninvasive imaging technique for staging hepatic fibrosis by assessing mechanical tissue properties [6–9]. It is based on the higher stiffness of fibrotic tissue induced by pathological changes such as the proliferation of collagen and cross-linking of free collagen branches [10]. Many currently available MRE techniques still suffer from limited anatomical resolution due to insufficient shear wave propagation and noise [11]. Tomoelastography by multifrequency MRE (MMRE) is a recently introduced advanced technique for generating full-field-of-view elastograms with pixel-wise detail resolution in a tomographic fashion [12]. It has been shown that tomoelastography outperformed previous elastograms generated by direct Helmholtz inversion in terms of detail resolution, noise robustness, and intra-tissue homogeneity [12]. With this technique, consistent mechanical tissue properties of small anatomical regions such as the spinal cord have been shown. So far, no diagnostic benefit of multifrequency over monofrequency MRE techniques has been established [13].

The primary aim of this study was to determine the diagnostic performance, cut-off values, and optimal drive frequency range for staging hepatic fibrosis using tomoelastography of the liver in patients with biopsy-proven fibrosis or imaging findings of cirrhosis. As a secondary aim, we investigated whether a combined analysis of the liver and spleen would further improve diagnostic performance.

## Materials and methods

### Subjects

**Fig. 1:**
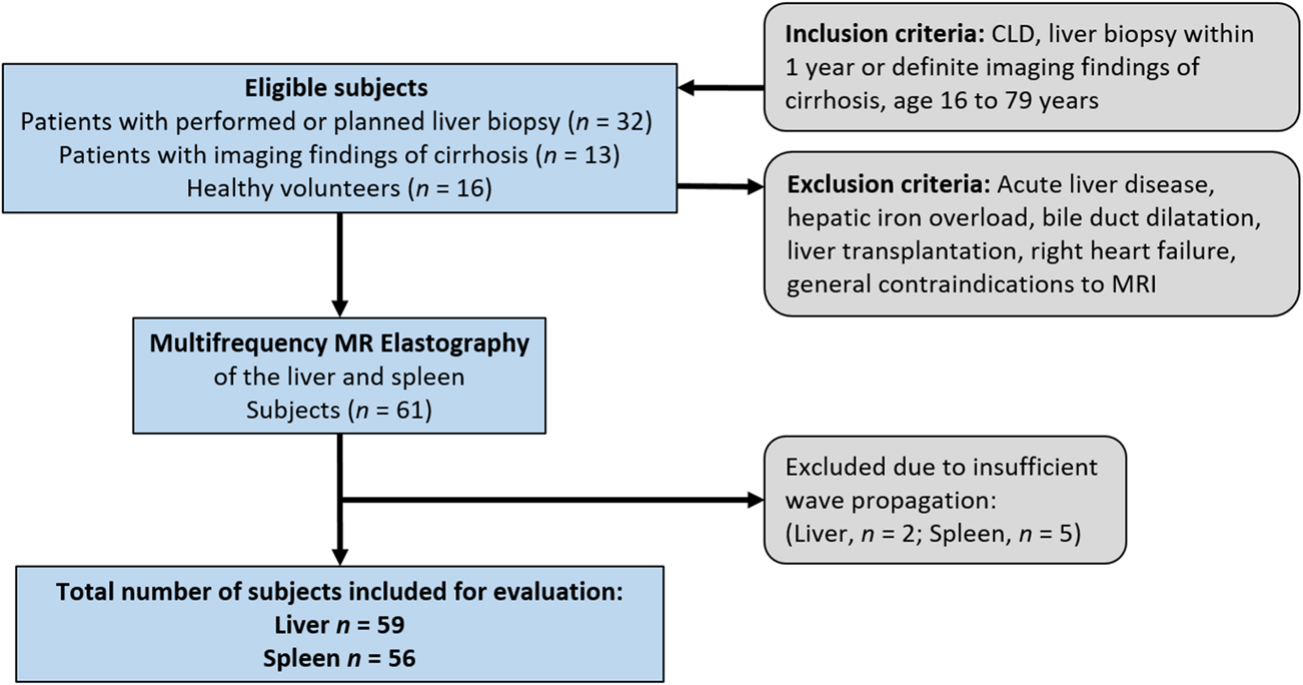
Flow diagram of study design and subjects. CLD, chronic liver disease

The study was approved by the institutional review board, and written informed consent was obtained from all subjects. In this prospective monocenter study, a total of 61 subjects were consecutively enrolled: 45 patients (18 women) and 16 healthy volunteers (8 women). Patients were examined between June 2014 and April 2017. Inclusion criteria were the presence of chronic liver disease (CLD) and liver biopsy performed or planned within 1 year of enrollment. Patients without biopsy but definite imaging findings of cirrhosis with a nodular liver contour and segmental hypertrophy or atrophy were also included since histological sampling would have been unethical in these cases. Further inclusion and exclusion criteria are shown in Fig. 1. All 16 healthy volunteers were characterized by ultrasound elastography (Virtual Touch Quantification, Acuson S2000, Siemens Healthineers) in a previous study to account for the absence of a histological reference standard [14]. Patients and healthy volunteers had a mean age of 49 years (range 16–75 years) and 52 years (range 31–75 years), respectively.

### Tomoelastography data acquisition

**Fig. 2:**
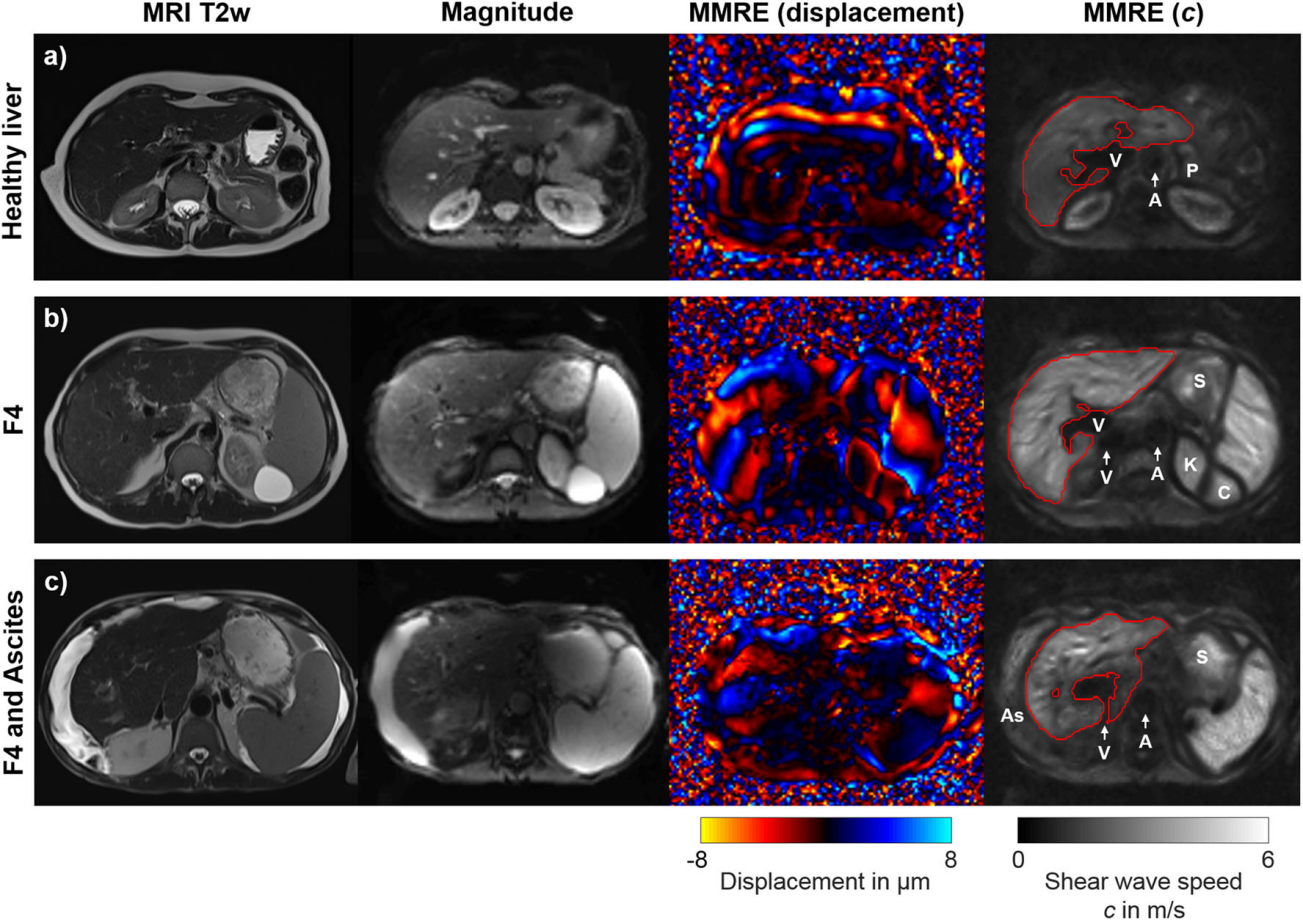
Tomoelastography of the upper abdomen. MRI T2w, conventional T2-weighted image with half-Fourier acquisition single-shot turbo spin echo (HASTE) sequence in axial orientation without contrast medium. Magnitude, morphological magnitude image derived from the multifrequency MR elastography (MMRE) sequence. MMRE (displacement), the shear wave image depicts tissue displacement in and out of the axial plane with red and blue colors. MMRE (*c*), compound multifrequency elastograms represent quantitative maps of shear wave speed (*c*) with bright and dark colors. Besides abdominal organs such as the liver, spleen, and kidneys, these maps also visualize smaller anatomical structures. The region of interest (red) indicates areas included in the analysis. V, hepatic vein/inferior vena cava; A, aorta; P, pancreas; K, kidney; S, stomach; C, kidney cyst; As, ascites. **a** A 61-year-old female healthy volunteer with mean *c* of 1.67 ± 0.25 m/s. **b** A 64-year-old male patient with chronic hepatitis C and cirrhosis (F4). Bright colors in the elastogram indicate a pathologically increased mean *c* of 2.59 ± 0.57 m/s. The left kidney (K) and a kidney cyst (C) are distinguishable from the spleen. **c** A 72-year-old male patient with chronic hepatitis C, cirrhosis (F4), and increased mean *c* of 2.46 ± 0.58 m/s. Shear wave propagation was not hindered by the presence of ascites (As)

Tomoelastography was performed on a 1.5 T MRI scanner (Magnetom Aera, Siemens Healthineers) with a 12-channel phased-array coil. We used a custom-designed piezoelectric driver, fast single-shot 3D wave-field acquisition at drive frequencies of 35 to 60 Hz with 5-Hz increments, as proposed by Hirsch et al [2]. Further imaging parameters were as follows: 9 slices, 8 time steps, 12 filter directions, 3 components, 78 × 100 matrix, 3 × 3 × 5 mm^3^ resolution, 2 averages, and 50-Hz motion-encoding gradient frequency. To avoid an increased postprandial hepatic blood flow and stiffness, subjects fasted for at least 4 h [15, 16]. The actuator was positioned at the level of the xiphoid process. Tomoelastography was performed in free breathing with a total image acquisition time of 4:30 min for all six frequencies combined. The total examination time was approximately 15–20 min and included patient and setup preparation, tomoelastography, and conventional MRI without contrast medium as follows: axial T1-weighted dual gradient-echo in-phase and out-of-phase sequence and axial and coronal T2-weighted half-Fourier acquisition single-shot turbo spin echo sequence. Examinations were performed by one of two radiologists with 5 (M.H.) and 7 (R.R.) years of experience in abdominal elastography. Image processing and evaluations were performed blinded to biopsy results by one observer (R.R.). Technical success of tomoelastography was evaluated by a visual assessment of the shear wave images (Fig. 2).

### Data processing

For image processing, we used a recently introduced tomoelastography pipeline, which is entirely available at https://bioqic-apps.charite.de and described in detail by Tzschätzsch et al [12]*.* Briefly, it has been demonstrated that complex wave number recovery and amplitude-weighted averaging of multiple harmonic frequencies in a compound map of stiffness outperforms detail resolution and noise robustness of single frequencies. Compound multifrequency processing improves elastograms by reducing areas of low shear wave displacement and wave nodes. Tomoelastography parameters are shear wave speed (*c* in m/s; *c* = wavelength × frequency) and penetration rate (*a* in m/s; *a* = penetration depth × frequency). While *c* relates to stiffness (the higher *c*, the stiffer the material), *a* relates to damping of shear waves (the higher *a*, the less attenuation is encountered). Full-field-of-view elastograms of the liver and spleen were derived from the same scan. Each slice of the elastograms was generated by compounding 216 images of MMRE raw data (12 spatiotemporal filter directions, 3 field components, 6 drive frequencies). Regions of interests were generated using a systematic approach: (i) The contours of liver and spleen were manually outlined using the magnitude image. (ii) A lower shear wave speed threshold of 1 m/s was applied to reduce boundary effects of major blood vessels and regions of insufficient shear wave excitation. Values above 1 m/s were included in the analysis. Consistent filter settings were used for all subjects. Liver fat content was calculated according to Fischer et al [17]. For patients, liver biopsy or imaging findings of cirrhosis were used as a reference standard. Histological fibrosis staging was performed according to Desmet et al [18].

### Statistical data analysis

The Shapiro–Wilk test was used to assess normal distribution with a level of significance of *p* ≤ 0.01. Spearman’s rank correlation coefficient (*R*_*s*_) was calculated for a pairwise comparison of all parameters with the stage of fibrosis. Pearson’s correlation coefficient (*R*_*p*_) was calculated for hepatic and splenic shear wave speed. A two-sided *t* test was used to assess the differences between patients and healthy volunteers as well as hepatic and splenic shear wave speed. The level of significance was *p* ≤ 0.05. Sensitivity, specificity, negative and positive predictive values, area under the receiver operating characteristic curve (AUC) with 95% confidence intervals (CI), and optimized cut-off values using the Youden index were calculated for fibrosis staging, accounting for single drive frequencies as well as compound multifrequency processing. AUC with 95% CI for combined hepatic and splenic parameters was calculated using binary logistic regression and compared with hepatic AUC with a level of significance of *p* ≤ 0.05, as described by DeLong et al [19]. Mean AUC values of all fibrosis stages combined were determined to compare single drive frequencies and compound multifrequency processing. A second observer (C.B.) reevaluated all cases and an interobserver reproducibility assessment was conducted by calculating the intraclass correlation coefficient (ICC) with 95% CI. Statistical analysis was conducted by an expert statistician (M.U.) using Matlab version 9.0 R2016a (The Mathworks, Inc.).

## Results

### Characteristics of subjects

The flow of subject enrollment is shown in Fig. 1. Mean values and standard deviation (SD) of body mass index (BMI) and liver fat content of patients and healthy volunteers were 25 ± 4 kg/m^2^ and 24 ± 4 kg/m^2^ and 3 ± 7% and 3 ± 5%, respectively. Liver fat content was not available for 1 patient. Healthy volunteers had no known history of any liver disease. Patients had the following CLDs: chronic hepatitis B and C (*n* = 6 and 5, respectively), primary sclerosing cholangitis (*n* = 9), nonalcoholic steatohepatitis (*n* = 6), autoimmune hepatitis (*n* = 6), toxic liver disease (*n* = 5), primary biliary cholangitis (*n* = 2), Wilson’s disease (*n* = 2), cryptogenic fibrosis (*n* = 2), diffuse liver metastases from breast cancer (*n* = 1), and alcoholic liver disease (*n* = 1). The mean (± SD) time interval between biopsy and tomoelastography was 69 ± 99 days. Fibrosis stage distribution in the patients included in the analysis (*n* = 43) based on the reference standard was as follows: F0, *n* = 1; F1, *n* = 10; F2, *n* = 5; F3, *n* = 9; and F4, *n* = 18. No correlation was found between age, BMI, liver fat content (*R*_*s*_ = 0.17, 0.04, − 0.12; with *p* = 0.19, 0.77, and 0.38, respectively) and the stage of fibrosis.

### Tomoelastography

**Fig. 3:**
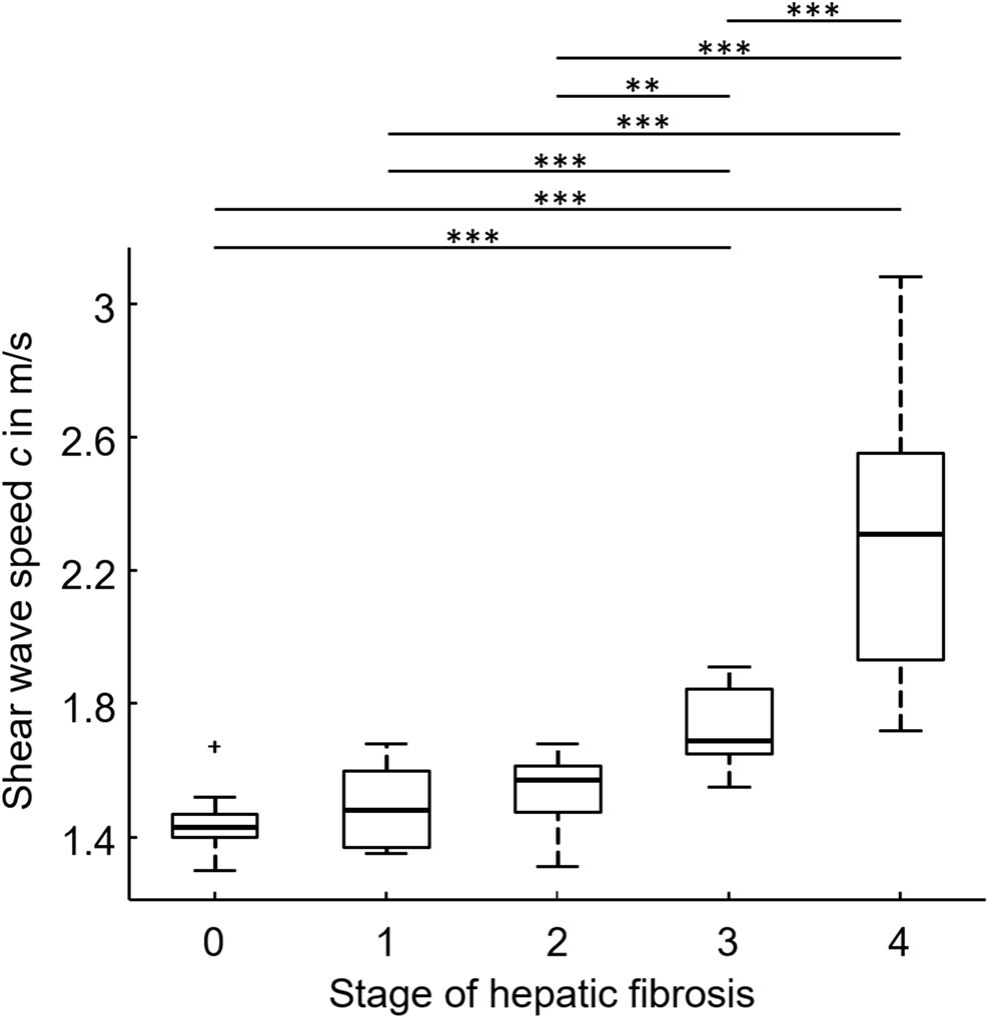
Boxplot of compound multifrequency (35–60 Hz) shear wave speed (*c*) of the liver and the stage of hepatic fibrosis. Median, upper, and lower quartile and whiskers of *c*-values are displayed. Statistically significant differences between groups of fibrosis stages are demarcated with asterisks: **p < 0.01; ***p < 0.001

Tomoelastography of the liver and spleen failed for 2 and 5 patients, respectively, due to insufficient shear wave propagation based on technical difficulties with the custom-designed piezoelectric driver setup. The overall technical success rate was 96.7% for the liver and 91.8% for the spleen. Figure 2 shows full-field-of-view elastograms with high detail resolution, even in patients with ascites. Besides the liver and spleen, also smaller anatomical structures such as the kidneys and kidney cysts, the pancreas, the aorta, and the portal vein and major hepatic veins are displayed in a tomographic fashion. The Shapiro–Wilk test showed normal distribution for all shear wave speed (*c*) data but not for penetration rate (*a*) data. Mean *c-* and *a*-values of the liver and spleen are listed in Table 1. Boxplots of hepatic *c*-values of compound multifrequency processing are displayed in Fig. 3. A significant difference in mean hepatic *c* derived from compound multifrequency processing was evident between patients and healthy volunteers (1.89 ± 0.44 m/s and 1.44 ± 0.08 m/s, respectively; *p* ≤ 0.0002); in contrast, no significant difference was found for mean splenic *c* (2.03 ± 0.56 m/s and 1.79 ± 0.36 m/s, respectively; *p* = 0.13). Mean *c* was significantly higher in the spleen compared with that in the liver for all single drive frequencies (all *p* ≤ 0.05) and compound multifrequency processing (*p* = 0.02). There was a strong significant correlation between hepatic *c* and the stage of fibrosis (Table 2). For splenic *c*, only a weak correlation with the stage of fibrosis was found, which was significant for all frequencies, except 35 Hz (*p* = 0.076; Table 2). For hepatic and splenic *c*, a weak to moderate significant correlation was evident, which became more pronounced towards higher frequencies (35 to 60 Hz with 5-Hz increments, and compound multifrequency: *R*_*p*_ = 0.32, 0.39, 0.46, 0.46, 0.46, 0.47, and 0.44, respectively; all with *p* ≤ 0.02). For liver fat content, there was a weak significant correlation with hepatic *a* (*R*_*p*_ = − 0.33, *p* = 0.01); in contrast, no significant correlation was found for hepatic *c* (*R*_*p*_ = − 0.18, *p* = 0.17).

**Table 1:** Mean shear wave speed (*c*) and penetration rate (*a*) values of the liver and spleen

Liver							Spleen					
Freq.	CTR / F0	F1	F2	F3	F4	All	CTR / F0	F1	F2	F3	F4	All
	(*n* = 17)	(*n* = 10)	(*n* = 5)	(*n* = 9)	(*n* = 18)	(*n* = 59)	(*n* = 17)	(*n* = 10)	(*n* = 5)	(*n* = 8)	(*n* = 16)	(*n* = 56)
Shear wave speed (*c*) in m/s												
35 Hz	1.42 (0.11)	1.45 (0.12)	1.45 (0.15)	1.64 (0.25)	1.87 (0.33)	1.60 (0.29)	1.58 (0.27)	1.73 (0.28)	1.68 (0.44)	1.61 (0.38)	1.94 (0.49)	1.72 (0.41)
40 Hz	1.44 (0.10)	1.48 (0.12)	1.52 (0.11)	1.69 (0.21)	2.10 (0.38)	1.69 (0.37)	1.71 (0.30)	1.85 (0.31)	1.75 (0.41)	1.72 (0.42)	2.15 (0.52)	1.87 (0.45)
45 Hz	1.45 (0.09)	1.52 (0.13)	1.57 (0.10)	1.72 (0.13)	2.32 (0.39)	1.78 (0.43)	1.81 (0.38)	1.97 (0.36)	1.90 (0.44)	1.76 (0.43)	2.39 (0.53)	2.00 (0.50)
50 Hz	1.47 (0.09)	1.57 (0.12)	1.57 (0.16)	1.78 (0.10)	2.47 (0.42)	1.85 (0.49)	1.92 (0.38)	2.02 (0.37)	1.98 (0.47)	1.84 (0.42)	2.52 (0.58)	2.10 (0.53)
55 Hz	1.49 (0.10)	1.61 (0.12)	1.60 (0.19)	1.80 (0.13)	2.53 (0.45)	1.88 (0.51)	2.05 (0.35)	2.10 (0.38)	2.02 (0.49)	1.89 (0.36)	2.62 (0.63)	2.19 (0.54)
60 Hz	1.52 (0.09)	1.63 (0.11)	1.66 (0.15)	1.83 (0.15)	2.59 (0.52)	1.92 (0.54)	2.07 (0.39)	2.14 (0.40)	2.09 (0.53)	1.91 (0.34)	2.73 (0.68)	2.25 (0.59)
35-60 Hz	1.44 (0.08)	1.50 (0.12)	1.54 (0.14)	1.73 (0.13)	2.30 (0.38)	1.77 (0.43)	1.78 (0.35)	1.90 (0.34)	1.85 (0.50)	1.69 (0.42)	2.37 (0.57)	1.96 (0.52)
Penetration rate (*a*) in m/s												
35 Hz	1.11 (0.08)	1.21 (0.06)	1.09 (0.09)	1.11 (0.12)	1.06 (0.10)	1.11 (0.10)	0.96 (0.11)	1.04 (0.10)	1.02 (0.09)	1.00 (0.09)	1.01 (0.11)	0.10 (0.11)
40 Hz	1.17 (0.10)	1.26 (0.09)	1.14 (0.10)	1.17 (0.13)	1.14 (0.11)	1.17 (0.11)	1.02 (0.12)	1.10 (0.11)	1.07 (0.14)	1.07 (0.14)	1.08 (0.14)	1.06 (0.13)
45 Hz	1.22 (0.11)	1.33 (0.09)	1.21 (0.11)	1.24 (0.15)	1.24 (0.12)	1.25 (0.12)	1.06 (0.14)	1.18 (0.12)	1.15 (0.17)	1.11 (0.21)	1.16 (0.17)	1.16 (0.17)
50 Hz	1.26 (0.12)	1.39 (0.10)	1.27 (0.15)	1.30 (0.16)	1.35 (0.13)	1.31 (0.13)	1.13 (0.15)	1.24 (0.14)	1.22 (0.21)	1.18 (0.27)	1.25 (0.22)	1.20 (0.20)
55 Hz	1.28 (0.12)	1.44 (0.12)	1.33 (0.20)	1.34 (0.16)	1.42 (0.14)	1.36 (0.15)	1.21 (0.18)	1.32 (0.17)	1.28 (0.25)	1.23 (0.31)	1.35 (0.27)	1.28 (0.24)
60 Hz	1.30 (0.12)	1.46 (0.14)	1.37 (0.22)	1.39 (0.17)	1.49 (0.15)	1.40 (0.16)	1.25 (0.19)	1.36 (0.16)	1.35 (0.27)	1.28 (0.34)	1.44 (0.29)	1.34 (0.26)
35–60 Hz	1.09 (0.08)	1.19 (0.06)	1.12 (0.13)	1.13 (0.11)	1.15 (0.08)	1.14 (0.09)	0.98 (0.13)	1.06 (0.12)	1.08 (0.19)	1.02 (0.26)	1.11 (0.20)	1.05 (0.19)

The F0 group includes 16 healthy volunteers (CTR, control group) and 1 patient with histology-proven stage F0. Numbers in parentheses are standard deviations. *Freq.*, frequency

**Table 2:** AUC values for staging hepatic fibrosis and correlation analysis

Freq.	AUC (95% CI)				Mean	Spearman	*p* value
	F ≥ 1	F ≥ 2	F ≥ 3	F4	AUC	Correlation	
Liver							
Shear wave speed (*c*)							
35 Hz	0.75 (0.64-0.85)	0.80 (0.69-0.90)	0.85 (0.75-0.94)	0.85 (0.75-0.94)	0.81	0.61	< 0.001*
40 Hz	0.82 (0.73-0.90)	0.89 (0.81-0.95)	0.92 (0.84-0.98)	0.95 (0.89-0.98)	0.89	0.76	< 0.001*
45 Hz	0.89 (0.81-0.96)	0.94 (0.89-0.99)	0.98 (0.94-1.00)	0.99 (0.97-1.00)	0.95	0.87	< 0.001*
50 Hz	0.89 (0.81-0.96)	0.94 (0.87-0.99)	0.98 (0.96-1.00)	0.99 (0.97-1.00)	0.95	0.87	< 0.001*
55 Hz	0.90 (0.83-0.96)	0.93 (0.86-0.98)	0.97 (0.94-1.00)	0.99 (0.97-1.00)	0.95	0.87	< 0.001*
60 Hz	0.92 (0.85-0.97)	0.93 (0.88-0.98)	0.97 (0.93-0.99)	0.98 (0.96-1.00)	0.95	0.87	< 0.001*
35–60 Hz	0.89 (0.81-0.95)	0.94 (0.89-0.99)	0.98 (0.96-1.00)	0.98 (0.96-1.00)	0.95	0.86	< 0.001*
Penetration rate (*a*)							
35 Hz	0.51 (0.39-0.64)	0.30 (0.19-0.41)	0.31 (0.20-0.43)	0.29 (0.18-0.42)	0.35	− 0.27	0.040*
40 Hz	0.51 (0.38-0.63)	0.35 (0.23-0.48)	0.39 (0.26-0.52)	0.38 (0.24-0.53)	0.41	− 0.17	0.207
45 Hz	0.60 (0.48-0.73)	0.44 (0.32-0.56)	0.47 (0.34-0.60)	0.48 (0.34-0.61)	0.50	0.01	0.928
50 Hz	0.69 (0.56-0.81)	0.53 (0.41-0.66)	0.54 (0.42-0.66)	0.58 (0.45-0.72)	0.59	0.19	0.158
55 Hz	0.73 (0.62-0.84)	0.58 (0.46-0.71)	0.60 (0.47-0.72)	0.64 (0.51-0.76)	0.64	0.29	0.028*
60 Hz	0.77 (0.65-0.88)	0.65 (0.54-0.76)	0.66 (0.55-0.79)	0.71 (0.59-0.82)	0.70	0.40	0.002*
35–60 Hz	0.71 (0.60-0.83)	0.53 (0.40-0.66)	0.54 (0.42-0.66)	0.56 (0.42-0.68)	0.59	0.19	0.157
Spleen							
Shear wave speed (*c*)							
35 Hz	0.63 (0.50-0.75)	0.58 (0.46-0.71)	0.59 (0.45-0.71)	0.68 (0.54-0.82)	0.62	0.24	0.076
40 Hz	0.63 (0.50-0.75)	0.60 (0.47-0.72)	0.63 (0.49-0.76)	0.73 (0.57-0.86)	0.65	0.29	0.030*
45 Hz	0.66 (0.51-0.78)	0.64 (0.52-0.76)	0.67 (0.55-0.79)	0.80 (0.68-0.91)	0.69	0.38	0.004*
50 Hz	0.64 (0.51-0.76)	0.64 (0.51-0.76)	0.66 (0.53-0.78)	0.80 (0.68-0.91)	0.68	0.36	0.006*
55 Hz	0.60 (0.47-0.71)	0.61 (0.47-0.74)	0.64 (0.50-0.76)	0.78 (0.65-0.90)	0.65	0.30	0.024*
60 Hz	0.62 (0.49-0.74)	0.63 (0.51-0.76)	0.65 (0.52-0.78)	0.80 (0.66-0.92)	0.68	0.35	0.009*
35–60 Hz	0.64 (0.51-0.75)	0.63 (0.49-0.75)	0.64 (0.51-0.76)	0.78 (0.65-0.90)	0.67	0.34	0.010*
Penetration rate (*a*)							
35 Hz	0.69 (0.55-0.82)	0.56 (0.43-0.69)	0.53 (0.40-0.66)	0.54 (0.40-0.68)	0.58	0.18	0.197
40 Hz	0.66 (0.53-0.80)	0.55 (0.43-0.68)	0.56 (0.42-0.68)	0.56 (0.42-0.69)	0.58	0.17	0.206
45 Hz	0.67 (0.54-0.80)	0.56 (0.42-0.68)	0.54 (0.40-0.66)	0.60 (0.47-0.73)	0.59	0.20	0.130
50 Hz	0.65 (0.52-0.78)	0.55 (0.42-0.68)	0.54 (0.41-0.67)	0.62 (0.46-0.76)	0.59	0.19	0.155
55 Hz	0.65 (0.51-0.77)	0.55 (0.42-0.67)	0.54 (0.41-0.67)	0.62 (0.49-0.75)	0.59	0.19	0.155
60 Hz	0.62 (0.49-0.74)	0.55 (0.42-0.68)	0.55 (0.42-0.69)	0.65 (0.49-0.79)	0.59	0.19	0.151
35–60 Hz	0.64 (0.50-0.76)	0.58 (0.45-0.71)	0.56 (0.43-0.69)	0.65 (0.50-0.79)	0.61	0.22	0.096

*P* values marked with an asterisk (*) indicate significant correlation (*p* < 0.05). AUC, area under the receiver operating characteristic curve; *CI*, confidence interval; *Freq.*, frequency

**Fig. 4:**
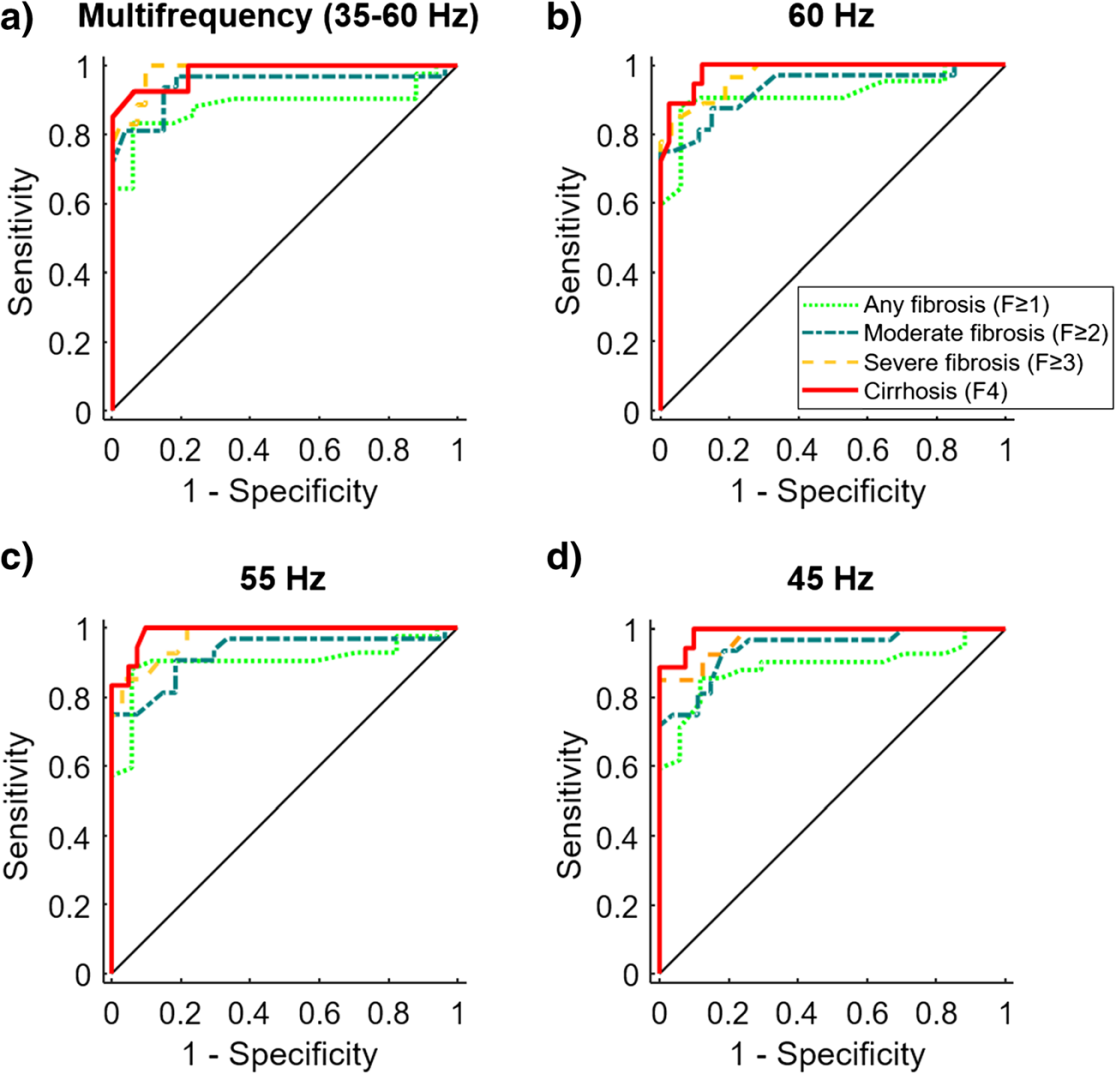
ROC curves for staging hepatic fibrosis based on shear wave speed (*c*) of the liver. Tomoelastography for the most important parameters: **a** compound multifrequency processing from 35–60 Hz as well as single drive frequencies of (**b**) 60 Hz, (**c**) 55 Hz, and (**d**) 40 Hz. Receiver operating characteristic (ROC) curves show values for any fibrosis (stage F1 or higher), moderate fibrosis (stage F2 or higher), severe fibrosis (stage F3 or higher), and cirrhosis (equivalent to stage F4)

AUC values with 95% CI and Spearman’s rank correlation coefficients of the liver and spleen as well as binary logistic regression analysis of the liver and spleen combined are compiled in Tables 2 and 3, respectively. Drive frequencies with the highest mean AUC and Spearman’s rank correlation coefficients were 45 Hz, 50 Hz, 55 Hz, 60 Hz, and compound multifrequency processing (all with mean AUC = 0.95, *R*_*s*_ ≥ 0.86 with *p* < 0.001; Table 2). In comparison, binary logistic regression analysis of combined hepatic and splenic *c*-values showed an increased mean AUC of 0.97 at 60 Hz; however, statistical significance was only evident for stage F4 (*p* < 0.03, Tables 3 and 4). Optimized diagnostic cut-off values of hepatic *c* with corresponding sensitivity, specificity, and negative and positive predictive values are shown in Table 5. Despite its high diagnostic accuracy, 50-Hz cut-off values failed in differentiating moderate fibrosis (F2) from severe fibrosis (F3) which limits clinical usefulness substantially. Receiver operating characteristic curves for the most important parameters (45 Hz, 55 Hz, 60 Hz, and compound multifrequency) are displayed in Fig. 4. For compound multifrequency processing of the liver, cut-off and AUC (with 95% CI) values were as follows: F1, 1.52 m/s and 0.89 (0.81–0.95); F2, 1.55 m/s and 0.94 (0.89–0.99); F3, 1.67 m/s and 0.98 (0.96–1.00); and F4, 1.72 m/s and 0.98 (0.96–1.00).

**Table 3:** AUC values for staging hepatic fibrosis using a combined analysis of liver and spleen

Binary logistic regression of liver and spleen					
Freq.	AUC (95% CI)				Mean
	F ≥ 1	F ≥ 2	F ≥ 3	F4	AUC
Shear wave speed (*c*)					
35 Hz	0.76 (0.64-0.89)	0.81 (0.70-0.93)	0.87 (0.77-0.97)	0.84 (0.71-0.97)	0.82
40 Hz	0.83 (0.72-0.94)	0.92 (0.84-1.00)	0.95 (0.89-1.02)	0.95 (0.87-1.03)	0.91
45 Hz	0.89 (0.80-0.97)	0.94 (0.88-1.01)	0.98 (0.94-1.02)	1.00 (0.98-1.02)	0.95
50 Hz	0.90 (0.82-0.98)	0.92 (0.85-1.00)	0.98 (0.95-1.02)	1.00 (0.97-1.02)	0.95
55 Hz	0.92 (0.85-0.99)	0.92 (0.85-1.00)	0.98 (0.94-1.02)	0.99 (0.96-1.02)	0.95
60 Hz	0.95 (0.90-1.00)	0.95 (0.90-1.00)	0.98 (0.94-1.02)	1.00 (0.98-1.02)	0.97
35–60 Hz	0.88 (0.79-0.97)	0.94 (0.87-1.00)	0.98 (0.94-1.02)	1.00 (0.98-1.02)	0.95
Penetration rate (*a*)					
35 Hz	0.70 (0.56-0.85)	0.74 (0.61-0.87)	0.71 (0.56-0.85)	0.80 (0.66-0.94)	0.74
40 Hz	0.70 (0.55-0.84)	0.73 (0.60-0.87)	0.68 (0.54-0.83)	0.74 (0.59-0.90)	0.71
45 Hz	0.66 (0.51-0.81)	0.67 (0.53-0.81)	0.61 (0.45-0.76)	0.68 (0.51-0.84)	0.66
50 Hz	0.69 (0.54-0.83)	0.56 (0.41-0.72)	0.54 (0.38-0.69)	0.61 (0.43-0.78)	0.60
55 Hz	0.62 (0.45-0.78)	0.58 (0.43-0.74)	0.60 (0.44-0.75)	0.64 (0.47-0.81)	0.61
60 Hz	0.78 (0.66-0.90)	0.65 (0.51-0.80)	0.68 (0.53-0.82)	0.70 (0.54-0.86)	0.70
35–60 Hz	0.72 (0.58-0.86)	0.58 (0.43-0.74)	0.56 (0.41-0.72)	0.66 (0.49-0.82)	0.63

*AUC*, area under the receiver operating characteristic curve; *CI*, confidence interval; *Freq.*, frequency

**Table 4:** *P* values for the comparison of two ROC curves: analysis of shear wave speed of liver and spleen versus liver alone

Freq.	*p* value			
	F ≥ 1	F ≥ 2	F ≥ 3	F4
35 Hz	0.90	0.98	1.00	0.97
40 Hz	0.99	0.86	0.94	0.78
45 Hz	0.91	0.98	0.94	0.70
50 Hz	0.82	0.84	0.81	0.78
55 Hz	0.80	0.93	0.67	0.86
60 Hz	0.49	0.70	0.67	0.03*
35–60 Hz	0.96	0.96	0.90	0.54

Statistically significant differences are demarcated with asterisks: *p < 0.05. *Freq.*, frequency; *ROC*, receiver operating characteristic curve

**Table 5:** Optimized cut-off values of hepatic shear wave speed (*c*) for staging fibrosis and corresponding sensitivity, specificity, and predictive values

Freq.	F ≥ 1					F ≥ 2					F ≥ 3					F4				
	Cut-off	Sn	Sp	NPV	PPV	Cut-off	Sn	Sp	NPV	PPV	Cut-off	Sn	Sp	NPV	PPV	Cut-off	Sn	Sp	NPV	PPV
	*c* in m/s					*c* in m/s					*c* in m/s					*c* in m/s				
35 Hz	1.47	0.69	0.82	0.52	0.91	1.57	0.66	0.89	0.69	0.88	1.59	0.78	0.91	0.83	0.88	1.59	0.83	0.78	0.91	0.63
40 Hz	1.50	0.76	0.82	0.58	0.91	1.67	0.72	0.96	0.74	0.96	1.68	0.85	0.97	0.89	0.96	1.68	0.94	0.83	0.97	0.71
45 Hz	1.51	0.86	0.88	0.71	0.95	1.56	0.94	0.82	0.92	0.86	1.71	0.85	1.00	0.89	1.00	1.75	1.00	0.90	1.00	0.82
50 Hz	1.59	0.81	0.94	0.67	0.97	1.73	0.78	1.00	0.79	1.00	1.73	0.93	0.94	0.94	0.93	1.85	0.94	0.95	0.98	0.90
55 Hz	1.58	0.88	0.94	0.76	0.97	1.74	0.75	1.00	0.77	1.00	1.78	0.85	0.97	0.89	0.96	1.83	1.00	0.90	1.00	0.82
60 Hz	1.62	0.88	0.94	0.76	0.97	1.78	0.75	1.00	0.77	1.00	1.82	0.85	0.97	0.89	0.96	1.85	1.00	0.88	1.00	0.78
35–60 Hz	1.52	0.83	0.94	0.70	0.97	1.55	0.94	0.85	0.92	0.88	1.67	0.93	0.94	0.94	0.93	1.72	1.00	0.90	1.00	0.82

*Freq.*, frequency; *Sn*, sensitivity; *Sp*, specificity; *NPV*, negative predictive value; *PPV*, positive predictive value; *c*, shear wave speed

For hepatic and splenic *a*, there was no consistent correlation with the stage of fibrosis, and diagnostic performance was poor with mean AUC values ranging from 0.35 to 0.70 (Table 2).

An excellent interobserver reproducibility with an ICC (95% CI) of 92% (85–96%) for the liver and 96% (93–98%) for the spleen was found.

## Discussion

To our knowledge, this is the first study investigating MRE of both the liver and spleen for staging hepatic fibrosis. We aimed to determine the diagnostic performance, cut-off values, and optimal drive frequency range of tomoelastography for this indication. For hepatic *c*, high AUC values suggest an excellent discriminative ability for staging hepatic fibrosis while detail resolution was improved compared with available MRE techniques. Full-field-of-view elastograms show a pixel-wise detail resolution in a tomographic fashion, which replaces the need to superimpose elastograms with conventional morphological images to identify abdominal organs. The best mechanical drive frequencies for the liver in terms of diagnostic performance are 45 Hz, 55 Hz, 60 Hz, and compound multifrequency processing.

Our results suggest a better diagnostic performance for higher drive frequencies and for staging severe fibrosis (F3) or cirrhosis (F4), which is consistent with the literature [7, 20]. For staging fibrosis at 60 Hz—the single drive frequency used in most studies—our results suggest cut-off and AUC values as follows: F1, 1.62 m/s and 0.92; F2, 1.78 m/s and 0.93; F3, 1.82 m/s and 0.97; and F4, 1.85 m/s and 0.98. Diagnostic performance is in the same range as reported by other studies [20–22]. A meta-analysis by Singh et al reported cut-off and AUC values as follows (cut-off values were transformed from kPa to m/s for better comparison): F1, 1.86 m/s and 0.84; F2, 1.91 m/s and 0.88; F3, 2.03 m/s and 0.93; and F4, 2.17 m/s and 0.92. Higher cut-off values and lower AUC values might be attributable to the combination of MRE techniques from various groups and to the investigation of a more diversified population [7]. Another meta-analysis by Singh et al, investigating the detection of liver fibrosis in patients with nonalcoholic fatty liver disease, found a similar performance with AUC values from F1 to F4 as follows: 0.86, 0.87, 0.90, and 0.91 [23].

Our current results demonstrate that the diagnostic performance of compound multifrequency processing is equivalent to that of higher single drive frequencies and not inferior as reported by Asbach et al [20]. Nevertheless, future studies could benefit from higher accuracy and shorter scan times when performing tomoelastography at higher frequencies only.

It is a stimulating result that the combined analysis of liver and spleen improved diagnostic performance in our study, which is in contrast to the results of an ultrasound elastography study by Leung et al [5]. However, the diagnostic benefit of combined elastography of the liver and spleen for fibrosis characterization strongly depends on the underlying systemic pathology, the presence of vascular obstructions, and portal hypertension. The fact that tomoelastography provides maps of the entire liver and spleen within a single scan will be of clinical relevance in many applications and renders MRE superior to complementary ultrasound-based elastography examinations.

For splenic *c* as well as hepatic and splenic *a*, low AUC values suggest a poor ability or failure to stage hepatic fibrosis. This poor sensitivity of *a*, as a representation of damping, for staging fibrosis has been shown previously [12, 20]. However, an even more pronounced significant correlation of hepatic *a* with steatosis has been demonstrated recently by a study investigating nonalcoholic fatty liver disease and should be implemented in future studies and data analysis for liver fat quantification [24]. Our results support their finding that hepatic damping increases with steatosis, although the liver fat content in our cohort was substantially lower.

Future studies should investigate the significance of tomoelastography for the assessment of focal liver lesions and fibrosis heterogeneity as an additional biomarker besides overall stiffness and compare the diagnostic performance of different MRE setups and image processing pipelines.

Our study has some limitations. First, we examined a small number of patients, especially in the F2 group, since liver biopsy is increasingly avoided in clinical routine in favor of noninvasive diagnostic tests. Moreover, we had a deviated population with a larger proportion of subjects in the F0 and F4 group, which can lead to overestimation of diagnostic performance. Second, there was a long interval between tomoelastography and biopsy, which can lead to misclassification. However, a recent meta-analysis suggests a low risk of disease progression bias when the interval is less than 1 year [7]. Third, as a monocenter study, data were acquired mainly from the same population, which favors overestimation of diagnostic performance. Fourth, fibrosis was caused by CLD of different etiologies. Fifth, for healthy volunteers, no biopsy was available as a reference test. Instead, healthy volunteers were assessed with an established ultrasound elastography method as a less reliable reference test. Sixth, we did not perform a reproducibility assessment. However, another study investigating the feasibility of tomoelastography of the prostate found a good overall test–retest reproducibility for this technique [25]. Finally, there was a technical success rate for liver and spleen of 96.7% and 91.8%, respectively. Examinations failed due to insufficient shear wave penetration and amplitudes. Compressed air drivers as used in [16, 26] have been proven a powerful alternative to our present piezo-based setup. This method was not available at the time of our study but will be implemented for future work.

In conclusion, tomoelastography provides cut-off values with excellent diagnostic accuracy for staging hepatic fibrosis. While diagnostic performance was comparable to that reported for other elastography techniques in prior studies, tomoelastography provided full-field-of-view elastograms of the abdomen with unprecedented pixel-wise detail resolution in a tomographic fashion. Our analysis of single-frequency tomoelastography suggests that scan time can be further reduced in future studies, making tomoelastography easier to implement in clinical routine.

## Abbreviations

AUC: Area under the receiver operating characteristic curve
BMI: Body mass index
CI: Confidence interval
CLD: Chronic liver disease
ICC: Intraclass correlation coefficient
MEG: Motion-encoding gradient
MMRE: Multifrequency magnetic resonance elastography
MRE: Magnetic resonance elastography
SD: Standard deviation

## References

[1] Guo J, Büning C, Schott E et al (2015) In vivo abdominal magnetic resonance elastography for the assessment of portal hypertension before and after transjugular intrahepatic portosystemic shunt implantation. Invest Radiol 50:347–351 10.1097/RLI.0000000000000136 25599282

[2] Hirsch S, Guo J, Reiter R (2014). MR elastography of the liver and the spleen using a piezoelectric driver, single-shot wave-field acquisition, and multifrequency dual parameter reconstruction. Magn Reson Med.

[3] Hirsch S, Guo J, Reiter R (2014). Towards compression-sensitive magnetic resonance elastography of the liver: sensitivity of harmonic volumetric strain to portal hypertension. J Magn Reson Imaging.

[4] Talwalkar JA, Yin M, Venkatesh S et al (2009) Feasibility of in vivo MR elastographic splenic stiffness measurements in the assessment of portal hypertension. AJR Am J Roentgenol 193:122–127 10.2214/AJR.07.3504 PMC2860633 19542403

[5] Leung VY, Shen J, Wong VW (2013). Quantitative elastography of liver fibrosis and spleen stiffness in chronic hepatitis B carriers: comparison of shear-wave elastography and transient elastography with liver biopsy correlation. Radiology.

[6] Horowitz JM, Kamel IR, Arif-Tiwari H (2017). ACR appropriateness criteria ® chronic liver disease. J Am Coll Radiol.

[7] Singh S, Venkatesh SK, Wang Z (2015). Diagnostic performance of magnetic resonance elastography in staging liver fibrosis: a systematic review and meta-analysis of individual participant data. Clin Gastroenterol Hepatol.

[8] Yin M, Talwalkar JA, Glaser KJ (2007). Assessment of hepatic fibrosis with magnetic resonance elastography. Clin Gastroenterol Hepatol.

[9] Huwart L, Sempoux C, Vicaut E (2008). Magnetic resonance elastography for the noninvasive staging of liver fibrosis. Gastroenterology.

[10] Reiter R, Freise C, Jöhrens K (2014). Wideband MRE and static mechanical indentation of human liver specimen: sensitivity of viscoelastic constants to the alteration of tissue structure in hepatic fibrosis. J Biomech.

[11] Hirsch S, Braun J, Sack I (2017) Magnetic resonance elastography, 1st edn. Wiley, Weinheim

[12] Tzschätzsch H, Guo J, Dittmann F (2016). Tomoelastography by multifrequency wave number recovery from time-harmonic propagating shear waves. Med Image Anal.

[13] Kennedy P, Wagner M, Castéra L (2018). Quantitative elastography methods in liver disease: current evidence and future directions. Radiology.

[14] Reiter R, Wetzel M, Hamesch K et al (2018) Comparison of non-invasive assessment of liver fibrosis in patients with alpha1-antitrypsin deficiency using magnetic resonance elastography (MRE), acoustic radiation force impulse (ARFI) Quantification, and 2D-shear wave elastography (2D-SWE). PLoS One. 10.1371/journal.pone.0196486 10.1371/journal.pone.0196486 PMC5919507 29698472

[15] Ipek-Ugay S, Tzschätzsch H, Hudert C (2016). Time harmonic elastography reveals sensitivity of liver stiffness to water ingestion. Ultrasound Med Biol.

[16] Dittmann F, Tzschätzsch H, Hirsch S (2017). Tomoelastography of the abdomen: tissue mechanical properties of the liver, spleen, kidney, and pancreas from single MR elastography scans at different hydration states. Magn Reson Med.

[17] Fischer MA, Raptis DA, Montani M (2012). Liver fat quantification by dual-echo MR imaging outperforms traditional histopathological analysis. Acad Radiol.

[18] Desmet VJ, Gerber M, Hoofnagle JH, Manns M, Scheuer PJ (1994) Classification of chronic hepatitis: diagnosis, grading and staging. Hepatology 19:1513–1520 8188183

[19] DeLong ER, DeLong DM, Clarke-Pearson DL (1988). Comparing the areas under two or more correlated receiver operating characteristic curves : a nonparametric approach. Biometrics.

[20] Asbach P, Klatt D, Schlosser B (2010). Viscoelasticity-based staging of hepatic fibrosis with multifrequency MR elastography. Radiology.

[21] Rustogi R, Horowitz J, Harmath C (2012). Accuracy of MR elastography and anatomic MR imaging features in the diagnosis of severe hepatic fibrosis and cirrhosis. J Magn Reson Imaging.

[22] Guo Y, Parthasarathy S, Goyal P, McCarthy RJ, Larson AC, Miller FH (2014) Magnetic resonance elastography and acoustic radiation force impulse for staging hepatic fibrosis: a meta-analysis. Abdom Imaging 40:818–834 10.1007/s00261-014-0137-6 24711064

[23] Singh S, Venkatesh SK, Loomba R (2016). Magnetic resonance elastography for staging liver fibrosis in non-alcoholic fatty liver disease: a diagnostic accuracy systematic review and individual participant data pooled analysis. Eur Radiol.

[24] Hudert CA, Tzschätzsch H, Rudolph B (2018). Tomoelastography for the evaluation of pediatric nonalcoholic fatty liver disease. Invest Radiol.

[25] Dittmann F, Reiter R, Guo J (2017). Tomoelastography of the prostate using multifrequency MR elastography and externally placed pressurized-air drivers. Magn Reson Med.

[26] Marticorena Garcia SR, Grossmann M, Bruns A (2019). Tomoelastography paired with T2* magnetic resonance imaging detects lupus nephritis with normal renal function. Invest Radiol.

